# 11β‐Hydroxysteroid dehydrogenase type 1 within muscle protects against the adverse effects of local inflammation

**DOI:** 10.1002/path.4806

**Published:** 2016-10-18

**Authors:** Rowan S Hardy, Craig L Doig, Zahrah Hussain, Mary O'Leary, Stuart A Morgan, Mark J Pearson, Amy Naylor, Simon W Jones, Andrew Filer, Paul M Stewart, Christopher D Buckley, Gareth G Lavery, Mark S Cooper, Karim Raza

**Affiliations:** ^1^ Institute of Inflammation and AgeingUniversity of BirminghamBirminghamUK; ^2^ Institute of Metabolism and Systems ResearchUniversity of BirminghamBirminghamUK; ^3^ Centre for Endocrinology Diabetes and MetabolismBirmingham Health PartnersEdgbastonBirminghamUK; ^4^ Faculty of Medicine and HealthSchool of MedicineUniversity of LeedsLeedsUK; ^5^ ANZAC Research InstituteUniversity of SydneySydneyAustralia; ^6^ Sandwell and West Birmingham Hospitals NHS TrustBirminghamUK

**Keywords:** muscle wasting, glucocorticoids, chronic inflammation, 11β‐HSD1, animal models

## Abstract

Muscle wasting is a common feature of inflammatory myopathies. Glucocorticoids (GCs), although effective at suppressing inflammation and inflammatory muscle loss, also cause myopathy with prolonged administration. 11β‐Hydroxysteroid dehydrogenase type 1 (11β‐HSD1) is a bidirectional GC‐activating enzyme that is potently upregulated by inflammation within mesenchymal‐derived tissues. We assessed the regulation of this enzyme with inflammation in muscle, and examined its functional impact on muscle. The expression of 11β‐HSD1 in response to proinflammatory stimuli was determined in a transgenic murine model of chronic inflammation (TNF‐Tg) driven by overexpression of tumour necrosis factor (TNF)‐α within tissues, including muscle. The inflammatory regulation and functional consequences of 11β‐HSD1 expression were examined in primary cultures of human and murine myotubes and human and murine muscle biopsies ex vivo. The contributions of 11β‐HSD1 to muscle inflammation and wasting were assessed in vivo with the TNF‐Tg mouse on an 11β‐HSD1 null background. 11β‐HSD1 was significantly upregulated within the tibialis anterior and quadriceps muscles from TNF‐Tg mice. In human and murine primary myotubes, 11β‐HSD1 expression and activity were significantly increased in response to the proinflammatory cytokine TNF‐α (mRNA, 7.6‐fold, p < 0.005; activity, 4.1‐fold, p < 0.005). Physiologically relevant levels of endogenous GCs activated by 11β‐HSD1 suppressed proinflammatory cytokine output (interkeukin‐6, TNF‐α, and interferon‐γ), but had little impact on markers of muscle wasting in human myotube cultures. TNF‐Tg mice on an 11β‐11β‐HSD1 knockout background developed greater muscle wasting than their TNF‐Tg counterparts (27.4% less; p < 0.005), with smaller compacted muscle fibres and increased proinflammatory gene expression relative to TNF‐Tg mice with normal 11β‐HSD1 activity. This study demonstrates that inflammatory stimuli upregulate 11β‐HSD1 expression and GC activation within muscle. Although concerns have been raised that excess levels of GCs may be detrimental to muscle, in this inflammatory TNF‐α‐driven model, local endogenous GC activation appears to be an important anti‐inflammatory response that protects against inflammatory muscle wasting in vivo. © 2016 The Authors. *The Journal of Pathology* published by John Wiley & Sons Ltd on behalf of Pathological Society of Great Britain and Ireland.

## Introduction

Glucocorticoids (GCs) are highly effective in the treatment of chronic inflammatory myopathies, controlling inflammation and protecting against muscle wasting and associated weakness 1. Similar beneficial effects are observed in patients with Duchenne muscular dystrophy, who develop progressive muscle wasting driven by muscle fibre necrosis, and in whom therapeutic GCs improve muscle strength and functional outcomes 2, 3, 4, 5, 6. The mechanisms behind these beneficial features of therapeutic GCs are not fully understood; however, inhibition of muscle inflammation, reduced fibre necrosis and increased myogenesis have been reported 7, 8, 9.

Unfortunately, prolonged exposure to therapeutic GCs is complicated by side effects, including myopathy that is mediated through inhibition of myogenesis, catabolic breakdown of muscle protein via suppression of insulin‐like growth factor 1 (IGF‐1), and increased proteolysis and atrophy of muscle fibres 10, 11, 12, 13. In many tissues, both the beneficial and detrimental effects of GCs have been explained by tissue‐specific targeting of their effects by 11β‐hydroxysteroid dehydrogenase type 1 (11β‐HSD1). This bidirectional enzyme primarily activates GCs [cortisone to cortisol in humans; 11‐dehydrocorticosterone (DHC) to corticosterone in mice] *in vivo*, and is a key determinant of exogenous GC effects in muscle, adipose tissue, liver and skin in mice 14.

11β‐HSD1 is present and biologically active in human skeletal muscle and in primary mesenchymal cell cultures derived from it, where it is implicated in GC‐induced muscle wasting 15, 16, 17, 18, 19, 20. We have previously demonstrated that mesenchymal cells, including osteoblasts, adipocytes, and fibroblasts, significantly upregulate 11β‐HSD1 expression in response to inflammation, greatly increasing local GC levels 21. This increase in local activation of physiological GCs exerts anti‐inflammatory effects, supporting the resolution of acute inflammation. However, during chronic inflammation, GCs generated by 11β‐HSD1 may be insufficient to drive normal resolution and, instead, persist at elevated levels, contributing to tissue damage 22.

The regulation and functional consequences of 11β‐HSD1 expression within muscle during inflammation have not yet been explored. This has particular relevance in chronic inflammation, where endogenous GC may possess a dual function, suppressing the effects of proinflammatory signalling and protecting against inflammatory myopathy, while augmenting mechanisms that mediate GC‐induced muscle wasting. We therefore evaluated the effects of 11β‐HSD1 activity on factors that regulate muscle physiology, and examined responses to inflammation *in vitro* and *in vivo*, by using a murine model of systemic inflammation in which the human proinflammatory cytokine tumour necrosis factor (TNF)‐α is expressed in all tissues, to dissect the relationship between local activation of physiologically relevant levels of GCs and inflammation.

## Materials and methods

### Human TNF‐α transgenic (TNF‐Tg) mouse model and clinical scoring

Experiments were performed according to strict guidelines governed by the UK Animal (Scientific Procedures) Act 1986 (project licence number 70/8582 or 70/8003), and were approved by the Birmingham Ethical Review Subcommittee. Tg197 mice (TNF‐Tg) were obtained from G Kollias (BSRC Fleming, Athens, Greece) 23. At 5–6 weeks of age, heterozygous TNF‐Tg mice develop spontaneous systemic chronic inflammation. Mice were scored three times weekly for body weight, paw swelling, and systemic inflammatory features. Clinical scores were calculated by scoring weight loss, behaviour, mobility, duration of joint swelling, mouse grimace, and evidence of joint inflammation (supplementary material, Table S1). At 9 weeks, mice were killed, and quadriceps and tibialis anterior muscle groups were collected. Heterozygous TNF‐Tg mice were crossed with mice with global deletion of 11β‐HSD1 to generate homozygous 11β‐HSD1 null mice that were heterozygous for the TNF‐Tg transgene 24.

### Primary human muscle biopsies

Following ethical approval (UK National Research Ethics Committee 14/ES/1044), patients with hip osteoarthritis (OA) (age 69 ± 3 years, Kellgren–Lawrence grade 3/4; *n* = 9) were recruited prior to elective joint replacement surgery. Between 150 and 200 mg of quadriceps muscle was collected at the time of surgery, and snap‐frozen in liquid nitrogen or collagenase‐digested to isolate myoblasts.

### Primary human muscle culture

Reagents were obtained from Sigma (Gillingham, UK) unless stated otherwise. Primary myoblasts (CC‐2580; Lonza, Slough, UK) were maintained in house in Skeletal Muscle Basal Medium‐2 (Lonza; CC‐3244 and CC‐3246) containing 0.1% human epidermal growth factor, 2% l‐glutamine, 10% fetal bovine serum (FBS) and 0.1% gentamicin/amphotericin‐B in the absence of GCs. Confluent myotubes were differentiated in Dulbecco's modified Eagle's medium (DMEM) containing 2% horse serum (HS) for 120 h. Media were replaced every 2–3 days 25.

### Primary murine muscle cell culture

Primary cultures of differentiated myotubes were generated from murine tibialis anterior muscles as previously described 26. In brief, whole tibialis anterior muscles were removed from wild‐type (WT) C57/Bl6 mice at 9 weeks, and digested by type 1 collagenase at 37 °C for 2 h before isolation of individual fibres. Fibres were plated in 2 ml of muscle expansion medium [DMEM High Glucose, 30% FBS, 10% HS, 1% chick embryo extract (CEE), and 10 ng/ml basic fibroblast growth factor] and grown in plates coated with Matrigel (Corning Life Sciences, Corning, NY, USA) (diluted 1:40 in DMEM High Glucose). Satellite cells migrating from muscle fibres were removed and cultured in maintenance medium (DMEM High Glucose 10% HS, 0.5% CEE) until confluent. Primary myoblasts were differentiated in selective medium (DMEM High Glucose, 2% HS) for 5 days until syncytialized myotubes formed.

### Analysis of mRNA abundance

The levels of expression of specific mRNAs were determined with TaqMan Gene Expression Assays (Thermo Fisher Scientific, Loughborough, UK). RNA was extracted from mature myotubes and muscle biopsies (50 mg) with a single‐step TRI Reagent extraction method. Aliquots (1 µg) of RNA were reverse transcribed by the use of random hexamers according to the manufacturer's protocol (4311235, Multiscribe; ThermoFisher Scientific). The levels of 11β‐HSD1 (*HSD11B1*), *H6PDH* and GRα (*NR3C1*) mRNAs were assessed to determine the expression of genes that impact on GC activation. *FBXO32*, *TRIM63* and *FOXO1* mRNAs were measured as markers of atrophy, *IGF1*, *IGF2* and *IRS1* mRNAs as anabolic markers, *MYOD1*, *MYF5*, *MYF6* and *MYOG* mRNAs as markers of differentiation, *MSTN* mRNA as a catabolic marker, and *GILZ*, *IL6* and *TNF* mRNAs as inflammatory markers. These and their murine counterparts were assessed with species‐specific probe sets by real‐time polymerase chain reaction (PCR) on an ABI7500 system (Applied Biosystems, Warrington, UK). Final reactions contained 2× TaqMan PCR mastermix (Thermo Fisher Scientific, Loughborough, UK), 200 nmol of TaqMan probe, and 25–50 ng of cDNA. The abundance of specific mRNAs in a sample was normalized to that of 18S RNA. Data were obtained as Ct values, and used to determine ΔCt values (Ct target – Ct 18S). Gene expression in arbitrary units (AU) was calculated from ΔCt values with the equation 1000 × −2ΔCt.

### Immunohistochemistry

Immunohistochemistry was performed on paraffin sections of human quadriceps muscle isolated from patients with OA, as previously described 27. In brief, sections were incubated with rabbit polyclonal antibodies against 11β‐HSD1 (1:50, 10007815, Lot no. 0452916; Cayman, Ann Arbor, Michigan, USA), and then with goat anti‐rabbit biotin as a secondary antibody (1:1000, AB360; The Binding Site, Birmingham, UK). Antigen retrieval was performed with 0.2 × trypsin in 1% (w/v) calcium chloride for 30 min at room temperature and blocking with 10% goat serum. Sections were incubated with horseradish peroxidase‐conjugated goat anti‐rabbit IgG (1:1000; Dako, Glostrup, Denmark) before visualization by incubation with 3,3′‐diaminobenzidine (Vector Laboratories, Burlingame, CA, USA) until development of a brown precipitate (5 min).

### Histological analysis of muscle

Histochemistry was performed on paraffin‐embedded 10‐µm sections of murine muscle biopsies isolated from the quadriceps muscles of WT, TNF‐Tg, 11β‐HSD1 knockout (11βKO) and TNF‐Tg/11βKO mice. Sections were stained with haematoxylin and eosin prior to quantitative analysis of fibre size and number in three 200‐µm^2^ regions of the vastus medialis from six mice per group with Image J software.

### 11β‐HSD1 activity

Confluent cells and tissues were cultured in medium containing either 100 nmol/l cortisone or 100 nmol/l DHC (to measure oxoreductase/activation activity) along with tritiated tracer amounts of cortisone or corticosterone (Perkin Elmer, Beaconsfield, UK). Steroids were extracted with dichloromethane and separated by thin‐layer chromatography with ethanol/chloroform (8:92) as the mobile phase. Thin‐layer chromatography plates were analysed with a Bioscan imager (Bioscan, Washington, DC, USA), and the fractional conversion of steroids was calculated. The protein concentration was determined with the Bio‐Rad Protein assay by use of the Bradford method (1‐800‐424‐6723; Bio‐Rad, Hercules, CA, USA), and sample concentrations were calculated from a known standard curve. Experiments were performed in triplicate, and results are expressed as pmol product per mg protein.

### Multiplex cytokine analysis

Levels of interleukin (IL)‐1β, IL‐1rα, IL‐4, IL‐8, IL‐10, IL‐17A, interferon (IFN)‐γ, monocyte chemotactic protein 1 (monocyte chemotactic and activating factor), macrophage inflammatory protein‐1α and TNF‐α in conditioned culture medium of primary human myotubes were measured simultaneously by the use of Bio‐Plex, Pro Human, Luminex MagPlex beads, according to the manufacturer's protocol (12001660 and 12001671; Bio‐Rad). In brief, culture medium was collected from primary human myotubes (generated as above) following treatment with either 10 ng/ml TNF‐α, 100 nmol/l cortisol, and 100 nmol/l cortisone, with or without 1 µmol/l LJ2 (PF‐877423). Samples were analysed with a Luminex plate reader and Starstation software (Applied Cytometry Systems, Sheffield, UK).

### MTT viability assay

CellTiter 96 (Promega, Southampton, UK) was used to determine the number of viable cells in murine and human primary myotube cultures (generated as above) following treatment with either the murine or human inactive GC precursor (100 nmol/l DHC or cortisone, respectively). Following 5 days in medium containing either vehicle control or inactive GC, cells were washed and incubated with medium containing 20% CellTiter 96 reagent. Plates were incubated at 37 °C for 50 min, after which absorbance was measured at 490 nm with a Victor3 multilabel counter (Perkin Elmer).

### Statistical analysis

Unless stated otherwise, data are shown as mean ± standard error (SE) of the mean for muscle biopsies and primary cultures derived from at least three independent mice or OA patients; animal studies were performed on six mice per experimental group (Table 1). Statistical significance was defined as a *p*‐value of <0.05 obtained with either an unpaired Student's *t*‐test or two‐way anova with Dunnett's *post hoc* analysis as appropriate. Throughout, sample sizes are presented as *N*, denoting numbers of independent mice and patients within a group, and *n*, denoting numbers of independent cell cultures derived from different donors. Statistical analysis of reverse transcription‐quantitative PCR (RT‐qPCR) data was carried out on ΔCt values.

**Table 1:** Numbers of samples assessed. (A) Numbers of osteoarthritis (OA) patients who donated muscle biopsies to the study, and the respective numbers in this cohort utilized in ex vivo biopsy and primary culture experiments. (B) Total numbers of mice per group [wild type (WT), tumour necrosis factor‐α transgenic (TNF‐Tg), 11β‐hydroxysteroid dehydrogenase type 1 knockout (11βKO), and TNF‐Tg/11βKO) utilized in ex vivo biopsy and primary culture experiments

	(A) OA patient quadriceps biopsies (total = 9)		(B) Mice per group (WT, TNF‐Tg, 11βKO, TNF‐Tg/11βKO) (total = 6)	
Tissue type	*Ex vivo* biopsy	Primary culture	*Ex vivo* biopsy	Primary culture
11β‐HSD1 activity	3	3	6	3
mRNA analysis	3	3	6	3
Histology	3	NA	6	NA
Cytokine analysis	NA	3	6	3
Fibre diameter	NA	3	6	3
Cell viability	NA	3	NA	3

NA, not applicable, as experiment not performed.

## Results

### TNF‐α‐driven chronic inflammation elevates 11β‐HSD1 expression at sites of inflammatory muscle wasting

The expression and activity of 11β‐HSD1 are upregulated at sites of inflammation 28, 29. However, relatively little is known about how this enzyme is regulated by inflammation within muscle. We examined 11β‐HSD1 expression and activity in muscles from a transgenic mouse model of chronic TNF‐α‐driven systemic inflammation (TNF‐Tg).

**Figure 1:**
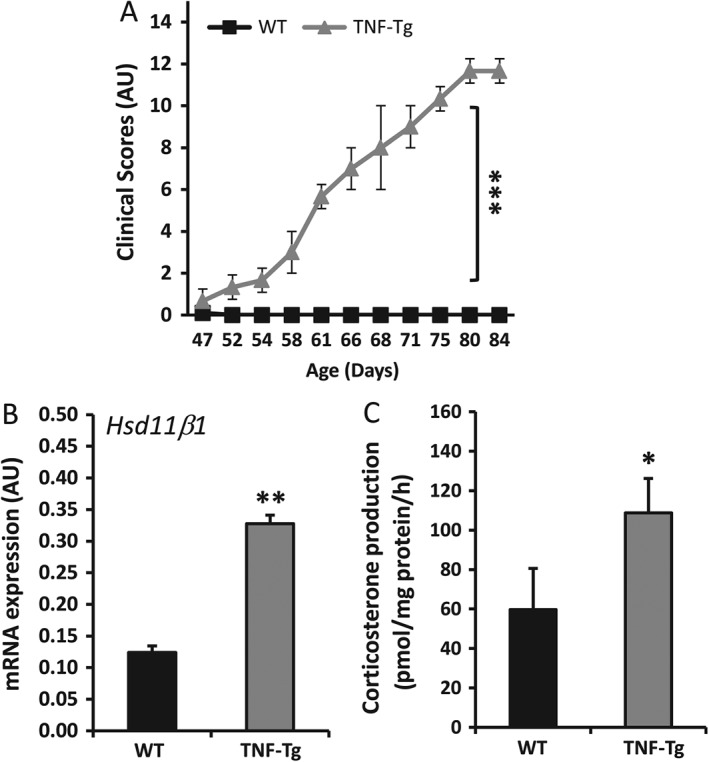
(A) Clinical scoring of behaviour, mobility, weight loss, mouse grimace, evidence of joint inflammation and duration of joint swelling (as outlined in supplementary material, Table S2) in TNF‐Tg and WT littermate controls between 5 and 9 weeks of age (N = 6 per group). (B) mRNA abundance (AU) of 11β‐HSD1 determined by RT‐qPCR in tibialis anterior muscles isolated from TNF‐Tg and WT controls at 9 weeks (N = 6 per group). (C) Rate of corticosterone generation by ex vivo biopsies of whole quadriceps muscles isolated from TNF‐Tg and WT controls following incubation for 16 h with DHC, determined by scanning thin‐layer chromatograms (N = 6 per group). Values are expressed as mean ± SE. Statistical significance was determined with Student's unpaired t‐test. *p < 0.05, **p < 0.005, ***p < 0.0005.

In this model, TNF‐α overexpression is driven via removal of the 3′‐untranslated region (UTR) from the *TNF* gene, and its subsequent replacement with the 3′‐UTR of the β‐globin gene (*HBB*), greatly increasing *TNF* mRNA transcriptional efficiency, stability and expression in all tissues, including muscle 23. At 5–6 weeks of age, TNF‐Tg mice develop a progressive systemic inflammatory phenotype, with paw swelling, polyarthritis, and weight loss (Figure 1A). In biopsies of tibialis anterior and quadriceps muscle, 11β‐HSD1 mRNA expression, and the ability to generate corticosterone from its inactive precursor DHC, were significantly increased in TNF‐Tg mice relative to WT littermate controls (mRNA, 2.7‐fold, *p* < 0.005; activity, 1.7‐fold, *p* < 0.05) (Figure 1B, C). These data provide the first evidence that 11β‐HSD1 expression is upregulated in muscle *in vivo* by TNF‐α‐driven inflammation.

### TNF‐α potently induces local cortisol generation in human muscle

To determine whether comparable inflammatory regulation of 11β‐HSD1 occurs within human muscle, we utilized biopsies of quadriceps muscles from OA patients, and also generated primary cultures of differentiated myotubes from human cells sourced commercially.

**Figure 2:**
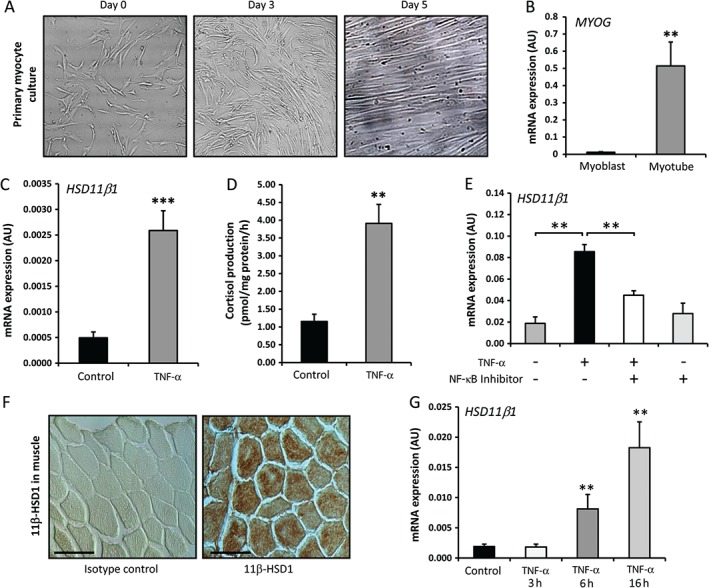
(A) Representative images of undifferentiated primary myoblasts isolated from human quadriceps. Primary myoblasts syncytialize over a 5‐day differentiation period in selective medium, forming primary myotubes in vitro. (B) Fold change in mRNA expression of the muscle differentiation gene MYOG in undifferentiated human myoblasts and mature mytotubes at day 5 after differentiation, determined by RT‐qPCR (n = 3 per group). (C, D) mRNA expression of the 11β‐HSD1 gene (C) and the rate of generation of cortisol from cortisone (D) in differentiated human myotubes over a period of 16 h following 24 h of pretreatment with TNF‐α (10 ng/ml), determined by RT‐qPCR and scanning thin layer chromatograms, respectively (n = 3 per group). (E) mRNA expression of the 11β‐HSD1 gene in differentiated human myotubes following treatment with TNF‐α (10 ng/ml) for 2 h in the presence or absence of the selective NF‐κB signalling inhibitor Mln‐4924 at 2 µm (n = 3 per variable). (F) Paraffin sections of quadriceps muscles isolated from OA patients were stained for 11β‐HSD1 by immunohistochemistry, and counterstained with Gill's haematoxylin (N = 3) (scale bars: 50 µm). (G) Gene expression (AU) of 11β‐HSD1 was measured by RT‐qPCR in ex vivo OA quadriceps biopsies following incubation with TNF‐α (10 ng/ml) for either 0, 3, 6 or 16 h (N = 3 per variable). Values are expressed as mean ± SE. Statistical significance was determined with using Student's unpaired t‐test (B–D) and one‐way anova with a Dunnett post hoc analysis (E, G). **p < 0.005, ***p < 0.0005.

Myoblasts were differentiated for 5 days in selective medium until cells underwent syncytialization, forming myotubes that spanned culture plates (Figure 2A). These were then cultured in maintenance medium for 72 h. Expression of the muscle differentiation marker myogenin was significantly increased in differentiated cultures (36‐fold relative to undifferentiated control, *p* < 0.0005) (Figure 2B). Incubation of fully differentiated primary myotubes with TNF‐α (10 mg/ml) resulted in significant increases in 11β‐HSD1 mRNA expression and cortisol synthesis (mRNA, 7.6‐fold, *p* < 0.005; activity, 4.1‐fold, *p* < 0.005) (Figure 2C, D). We have previously demonstrated that TNF‐α regulates 11β‐HSD1 in mesenchymal‐derived cells via nuclear factor‐κB (NF‐κB) signalling. Therefore, we examined this pathway in in myotubes by using the inhibitor of NF‐κB signalling Mln‐4924 (2 µm) 30. Whereas 11β‐HSD1 mRNA expression was strongly induced at 2 h by 10 ng/ml TNF‐α (five‐fold increase, *p* < 0.005), this induction was abrogated by the selective NF‐κB inhibitor (Figure 2E). Thus, the induction of 11β‐HSD1 by TNF‐α in muscle is regulated through the NF‐κB pathway.

Next, we examined the basal expression of 11β‐HSD1 and its regulation by TNF‐α in muscle biopsies from OA patients. 11β‐HSD1 was expressed throughout human muscle fibres (Figure 2F). To better delineate the relevance of *in vitro* findings to muscle *ex vivo*, we examined 11β‐HSD1 expression and its inflammatory regulation by TNF‐α by using quadriceps muscle biopsies (Figure 2G). Basal mRNA expression of 11β‐HSD1 was observed, with significant induction by TNF‐α (10 ng/ml) at 6 and 16 h [4.2‐fold (*p* < 0.005) and 9.6‐fold (*p* < 0.005) increase relative to samples at baseline, respectively]. Therefore, in both human myotube cultures and in *ex vivo* human muscle biopsies, 11β‐HSD1 was potently upregulated by TNF‐α. This suggests that human muscle has the capacity to respond to acute inflammatory stressors by increasing local GC production. Next, we assessed the significance and functional consequences of elevated endogenous GC production within muscle during inflammation.

### Locally activated GCs suppress proinflammatory cytokine production in primary myotube culture

Given the upregulation of 11β‐HSD1 expression and activity within muscle following inflammatory stimulation, we assessed the functional consequences of raised intramuscular endogenous GC levels in primary myotube cultures.

**Figure 3:**
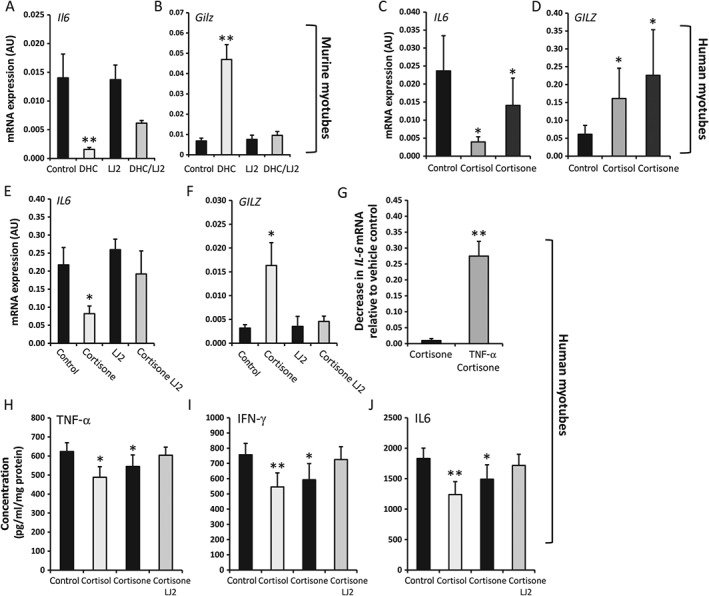
(A, B) Gene expression (AU) of Il6 and Gilz in murine primary cultures of differentiated myotubes isolated from WT quadriceps biopsies (n = 3 per variable). Cultures were incubated for 16 h with either the inactive corticosterone precursor DHC (100 nmol/l), the selective 11β‐HSD1 inhibitor LJ2 (1000 nmol/l), or a combination of the two. (C–G) Gene expression (AU) of IL6 and GILZ in human primary cultures of differentiated myotubes isolated from quadriceps muscles (n = 3 per variable). Cultures were stimulated with either vehicle (C, D) or TNF‐α (E, F) at 10 ng/ml for 48 h (n = 3 per variable). Cultures were then maintained in medium containing either vehicle control, cortisol (100 nmol/l), the inactive corticol precursor cortisone (100 nmol/l), the selective 11β‐HSD1 inhibitor LJ2 (1000 nmol/l) or a combination of both cortisone and inhibitor for 16 h. (G), The decrease in IL6 mRNA level relative to respective vehicle controls following incubation with cortisone in the vehicle and TNF‐α‐prestimulated groups was then measured (n = 3 per variable). (H–J) Concentrations of IL‐6, TNF‐α and IFN‐γ within 48‐h conditioned medium of primary human myotubes treated with either control, cortisol (100 nmol/l), cortisone (100 nmol/l), or LJ2 (1000 nmol/l) and cortisone, determined by multiplex cytokine analysis (n = 3 per variable). Values are expressed as mean ± SE. Statistical significance was determined with one‐way anova with a Dunnett post hoc analysis. *p < 0.05, **p < 0.005. LJ2 = PF‐877423.

IL‐6 was examined as a cytokine that is potently upregulated in response to TNF‐α in mesenchymal‐derived cells. In murine muscle culture, the endogenous corticosterone precursor DHC strongly reduced *IL6* mRNA levels (9.3‐fold, *p* < 0.005) (Figure 3A), whereas expression of the anti‐inflammatory GC response gene *GILZ* was significantly increased (Figure 3B). These effects were abrogated by the selective 11β‐HSD1 inhibitor LJ2. Similar observations were made in primary human myotube culture, with the inactive precursor cortisone having similar effects on both *IL6* and *GILZ* mRNA expression as the active GC cortisol (*IL6*, 7.1‐fold decrease, *p* < 0.005; *GILZ*, 2.6‐fold increase, *p* < 0.005) (Figure 3C, D). When primary human myotube cultures were preincubated with TNF‐α to induce 11β‐HSD1 activity, similar patterns of *IL6* and *GILZ* expression were found in response to cortisone (Figure 3E, F). However, the absolute cortisone suppression of *IL6* mRNA was significantly greater in TNF‐α‐prestimulated myotubes than in vehicle‐treated controls (30‐fold greater suppression of *IL6* in TNF‐α‐prestimulated myotubes; *p* < 0.0005) (Figure 3G). The effects of cortisone were entirely blocked in the presence of the selective 11β‐HSD1 inhibitor LJ2 in the TNF‐α‐pretreated human myotube culture (Figure 3E, F).

We then examined the cytokine profiles of primary human myotubes following incubation with cortisone (Figure 3H–J). The expression levels of the proinflammatory cytokines IL‐6, TNF‐α and IFN‐γ were all significantly reduced following treatment with cortisone (IL‐6, 18.6% decrease, *p* < 0.05; TNF‐α, 12.7% decrease, *p* < 0.05; IFN‐γ, 21% decrease, *p* < 0.05); these effects were abrogated by the selective 11β‐HSD1 inhibitor LJ2.

For the first time, we have demonstrated that upregulation of 11β‐HSD1 by TNF‐α influences the inflammatory profiles of both murine and human primary myotubes, suppressing proinflammatory cytokine output and enhancing GILZ signalling.

### Locally activated GCs suppress anabolic and induce catabolic pathways in murine, but not human, muscle culture

**Figure 4:**
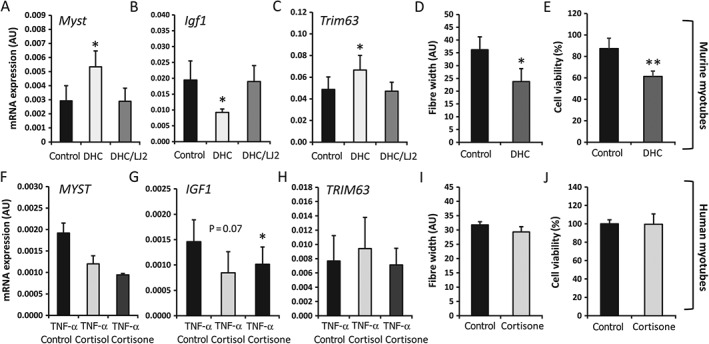
Levels of mRNA in primary cultures of differentiated myotubes isolated from quadriceps muscle biopsies from WT mice (A–C) and human quadriceps biopsies (F–H) (n = 3 per variable), determined by RT‐qPCR. To induce 11β‐HSD1 expression, human primary cultures were pretreated with TNF‐α (10 ng/ml) for 48 h prior to a 12‐h washout. Cells were then incubated for 16 h with either control, active cortisol (100 nmol/l), or its inactive precursor cortisone (100 nmol/l). In murine culture, cells were treated for 24 h with either control medium, the inactive corticosterone precursor DHC (100 nmol/l), or DHC in combination with the selective 11β‐HSD1 inhibitor LJ2 (1000 nmol/l). Fibre width and cell viability were determined with Image J analysis software and MTT assay, respectively, in murine (D, E) and human (I, J) primary myotubes following incubation with normal control medium and DHC (100 nmol/l)‐containing medium over a period of 5 days (n = 3 per variable). Values are expressed as mean ± SE. Statistical significance was determined with one‐way anova with a Dunnett post hoc analysis. *p < 0.05, **p < 0.005.

Previous studies have shown that prolonged exposure to supraphysiological levels of GCs results in muscle wasting *in vivo* and increased atrophy of myotubes and catabolic breakdown of protein *in vitro*. Consequently, we examined the effects of endogenous GCs on mRNA levels of genes central to these processes, including *MYOG*, *IGF1*, and *FOXO1*. In murine primary myotubes, expression of the catabolic factor gene *Mstn* was significantly increased (1.8‐fold, *p* < 0.05), that of the anabolic signalling factor gene *Igf1* was significantly suppressed (1.37‐fold, *p* < 0.05) and that of the atrophy marker gene *Trim63* was significantly increased (1.4‐fold, *p* < 0.05) by the GC precursor DHC at 100 nmol/l (Figure 4A–C). These effects were abrogated by selective 11β‐HSD1 inhibition. Analysis of a panel of differentiation, anabolic, catabolic and atrophy genes, including *Myog*, *Myod1*, *Foxo1*, *Fbxo32*, *Trim63*, *Igf1*, and *Igf2*, revealed no additional genes that were responsive to endogenous levels of GCs (supplementary material, Figure S2). Exposure to DHC for 5 days resulted in a significant reduction in murine myotube fibre size and reduced cell viability (Figure 4D, E). These effects were not observed in primary human myotubes exposed to cortisone, either in the absence of TNF‐α or after induction of 11β‐HSD1 by TNF‐α (Figure 4F–J; supplementary material, Figure S1).

These data provide evidence that locally activated endogenous GCs increase muscle wasting through the 11β‐HSD1 enzyme in murine muscle *in vitro*. In contrast, the actions of endogenous GCs were less conclusive in the human *in vitro* model, with no functional evidence that they mediate muscle wasting being obtained.

### Global deletion of 11β‐HSD1 results in pronounced muscle wasting in the TNF‐Tg mouse

To delineate the contribution of inflammatory 11β‐HSD1 expression and GC activation to muscle inflammation and muscle wasting *in vivo*, we generated a TNF‐α‐driven murine model (TNF‐Tg) of muscle wasting on a global 11β‐HSD1 null (TNF‐Tg/11βKO) background.

**Figure 5:**
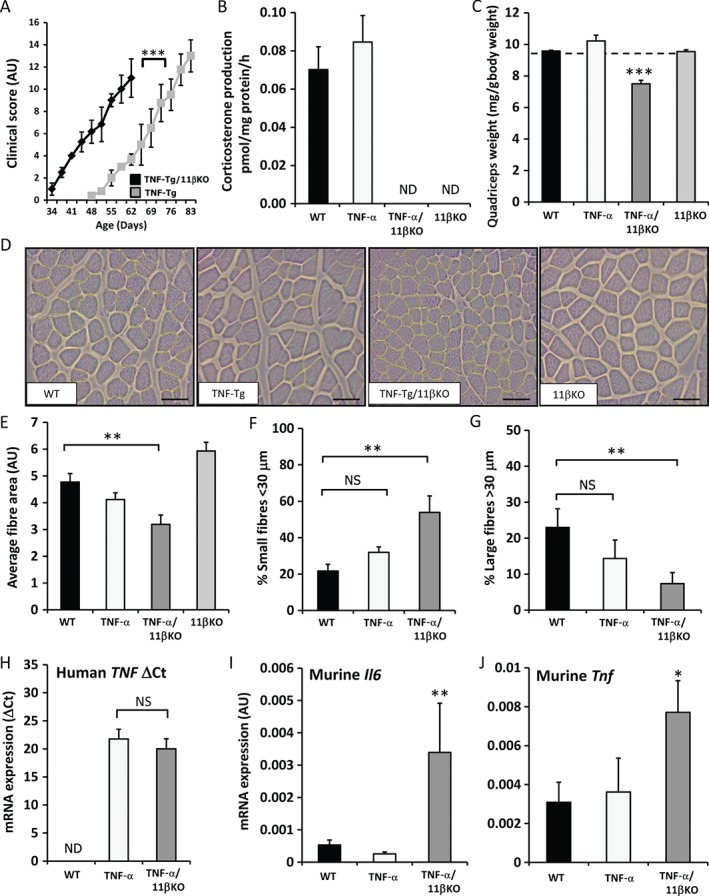
(A) Clinical scoring of behaviour, mobility, weight loss, mouse grimace, evidence of joint inflammation and duration of joint swelling (as outlined in supplementary material, Table S2) in TNF‐Tg/11βKO mice versus TNF‐Tg controls between 5 and 9 weeks of age (N = 6 per group). (B) Rate of corticosterone generation by ex vivo biopsies of whole quadriceps muscles isolated from WT, TNF‐Tg, 11βKO and TNF‐Tg mice on an 11βKO background following incubation for 16 h with DHC, as determined by scanning thin‐layer chromatograms (N = 6 per group). (C) Total quadriceps muscle weight normalized to total body weight in WT, TNF‐Tg, 11βKO and TNF‐Tg mice on an 11βKO background (N = 6 per group). (D) Representative images of quadriceps muscle sections taken from WT and 11βKO mice on either control or TNF‐Tg backgrounds stained with Gill's haematoxylin and eosin (scale bars: 50 µm) (N = 6 per group). (E–G) Average fibre size (AU), and quantification of small (<30 µm) and large (≥20 µm) fibres (AU) determined with Image J in paraffin sections of quadriceps muscles isolated from WT mice, TNF‐Tg mice, and TNF‐Tg mice on an 11βKO background (N = 6 per group). (H–J) Levels of mRNA (ΔCt) for human TNF‐α and the murine proinflammatory genes Il6 and Tnf in WT mice, TNF‐Tg mice and TNF‐Tg mice on an 11βKO background at 9 weeks (N = 6 per group). Values are expressed as mean ± SE. Statistical significance was determined with one‐way anova with a Dunnett post hoc analysis. ND, not detected; NS, not significant. *p < 0.05, **p < 0.005, ***p < 0.0005.

TNF‐Tg/11βKO mice presented with a more rapid onset of systemic inflammation than TNF‐Tg mice (Figure 5A). Examination of quadriceps and tibialis anterior muscles confirmed a complete absence of endogenous 11β‐HSD1‐mediated GC activation in TNF‐Tg/11βKO mice (Figure 5B). When normalized for body weight, TNF‐Tg/11βKO mice had significantly reduced quadriceps weights relative to age‐matched TNF‐Tg mice (TNF‐Tg, 10.2 ± 0.75 mg/g total body weight; TNF‐Tg/11βKO, 7.49 ± 0.43 mg/g total body weight; *p* < 0.0005) (Figure 5C). Histological analysis of muscle in TNF‐Tg/11βKO mice revealed smaller fibres than those from their TNF‐Tg, 11βKO and WT counterparts (Figure 5D). No significant inflammatory infiltrates were evident within muscle biopsies from TNF‐Tg or TNF‐Tg/11βKO mice, indicating this phenotype was not underpinned by invading leukocytes. Examination of muscle fibres in TNF‐Tg/11βKO mice confirmed that the average fibre diameter was significantly reduced relative to TNF‐Tg mice (3.1 ± 0.26 AU versus 4.1 ± 0.21 AU, respectively; *p* < 0.005), driven by an increase in the number of small fibres and a reduction in the number of large fibres (Figure 5E–G). The number of fibres with centralized nuclei was consistent between groups, indicating that the phenotype was not driven by increased generation of fibres, as is observed in conditions of increased muscle turnover and repair. Instead, these data suggest increased inflammatory wasting in TNF‐Tg/11βKO mice relative to their TNF‐Tg and WT counterparts. Examination of anabolic, catabolic, differentiation and muscle atrophy genes (supplementary material, Figure S2) failed to identify significant changes between TNF‐Tg and TNF‐Tg/11βKO mice, despite elevated expression of human TNF‐α mRNA within muscle (Figure 5H). In contrast, gene expression levels of both endogenous murine TNF‐α and IL‐6 were markedly increased in TNF‐Tg/11βKO mice relative to their TNF‐Tg counterparts (IL‐6, 13.2‐fold, *p* < 0.005; TNF‐α, 2.1‐fold, *p* < 0.05) (Figure 5I, J). Consequently, given the increased severity of the wasting phenotype in the absence of 11β‐HSD1, this model suggests that, instead of contributing to inflammatory muscle wasting, endogenously generated GCs protect against the deleterious effects of excess inflammation in muscle.

## Discussion

The actions of therapeutic and endogenous GCs are closely regulated by the tissue‐specific actions of the GC‐activating enzyme 11β‐HSD1. We examined the role of 11β‐HSD1 in muscle during inflammation, and assessed whether increased activation of physiologically relevant levels of endogenous GCs influences inflammation associated muscle loss.

We have previously reported that 11β‐HSD1 activity is potently upregulated by proinflammatory cytokines such as TNF‐α and IL‐1β within mesenchymal cells, such as osteoblasts, adipocytes, dermal fibroblasts, and stromal fibroblasts 21, 22, 29, 31. In this study, we demonstrate that 11β‐HSD1 activity is potently upregulated through the NF‐κB signalling pathway by TNF‐α in human primary muscle cultures, in human muscle *ex vivo*, and *in vivo* in a TNF‐α‐driven murine model of chronic inflammation and muscle wasting. Given the importance of 11β‐HSD1 in mediating tissue‐specific responsiveness to GCs, the marked upregulation with inflammation raises important questions regarding its role in muscle during inflammation.

In the context of non‐muscle cells, such as synovial fibroblasts, we previously reported that GCs activated by 11β‐HSD1 suppress proinflammatory signalling 22, 29. Furthermore, 11β‐HSD1 is critical in mediating the resolution of inflammation in murine models of peritonitis and acute polyarthritis 22, 32, 33. Despite these protective anti‐inflammatory actions, GCs contribute to muscle wasting when administered in excess. Consequently, we focused on how, within muscle, the endogenous physiologically relevant levels of GCs activated by 11β‐HSD1 influence markers of muscle metabolism, atrophy and differentiation, and inflammation.

We found, as previously reported in murine myotube culture, that extended exposure to endogenous GCs activated by 11β‐HSD1 resulted in a shift in IGF‐1 and myostatin gene expression, favouring catabolic muscle breakdown. This correlated with increased levels of markers of atrophy, and resulted in decreased myotube fibre size and viability. However, unlike murine myotubes, primary human myotube cultures were relatively resistant to the actions of physiologically relevant concentrations of GCs generated by 11β‐HSD1, with there being no marked change in metabolic, atrophy or differentiation genes either in the presence or in the absence of TNF‐α. It is important to note that the translational relevance of murine models to human disease is often contested. In this study, we observed differences in endogenous GC action on muscle wasting between primary human and murine muscle cultures. This indicates a need for further characterization of these differences in future studies utilizing these *in vitro* models.

The actions of proinflammatory cytokines and the NF‐κB pathway have been shown to be integral to inflammatory muscle wasting, mediated in part through their regulation of the ubiquitin proteasome system 34, 35, 36, 37, 38. Many of the anti‐inflammatory actions of GCs are mediated through suppression of signalling pathways such as AP‐1 and NF‐κB, resulting in the suppression of downstream inflammatory mediators 39, 40. When we examined markers of proinflammatory signalling, we observed that endogenous GCs activated by 11β‐HSD1 potently suppressed IL‐6, IFN‐γ and TNF‐α expression in both primary murine and human muscle culture. Given the known role of these proinflammatory cytokines in the inflammatory muscle wasting process, the effects of GCs generated by 11β‐HSD1 were seen to be overwhelmingly anti‐inflammatory and potentially muscle‐sparing.

Taken together, these findings indicate dual effects of endogenous GCs generated by 11β‐HSD1 during inflammation; although they drive muscle wasting via well‐defined GC‐mediated wasting pathways, 11β‐HSD1 also mediates potent anti‐inflammatory effects of GCs that may be muscle‐sparing. To ascertain which of these 11β‐HSD1‐mediated effects predominates in muscle *in vivo*, in the context of chronic inflammatory muscle wasting, we utilized the TNF‐Tg transgenic mouse (TNF‐Tg), on an 11β‐HSD1 global null background (TNF/11βKO). Having demonstrated that human TNF‐α is upregulated to a similar extent in both the TNF‐Tg and TNF/11βKO mice, we explored whether TNF/11βKO mice would be spared 11β‐HSD1‐mediated GC muscle wasting during muscle inflammation, or whether the loss of inflammation suppression by 11β‐HSD1‐generated GCs would exacerbate inflammatory muscle loss. In our model, the absence of endogenous GC generation by 11β‐HSD1 in the TNF‐Tg mouse did not abrogate or reverse inflammatory muscle wasting. Instead, these mice developed significantly greater muscle loss, with smaller muscles and muscle fibres.

These effects correlated with significantly higher levels of proinflammatory gene expression in muscle than in TNF‐Tg controls in the absence of infiltrating leukocytes. Given the well‐recognized role of these proinflammatory cytokines in mediating muscle wasting, it is likely that the loss of their suppression, in the absence of 11β‐HSD1, exacerbates inflammatory muscle wasting. Although increased leukocyte infiltration driven by suppression of 11β‐HSD1 within muscle can be ruled out as a primary factor, it should be noted that, given the global nature of this null model, changes in inflammatory output by immune cells at distant locations or in local stromal populations cannot be ruled out as contributing to this wasting phenotype. Although it has been reported that endogenous GCs play a role in the differentiation of mesenchymal‐derived cells such as myoblasts, we observed no shift in the expression of differentiation markers from TNF‐Tg/11βKO mice that would underpin this phenotype through reduced myogenesis (supplementary material, Figure S2) 41. Furthermore, 11βKO mice retain a normal muscle phenotype without evidence of reduced myoblast differentiation, suggesting that circulating GCs are sufficient for basal muscle differentiation in these animals 42. Consequently, these data suggest a more important role for 11β‐HSD1 in modifying inflammation within muscle, protecting against muscle wasting by suppressing the expression of proinflammatory cytokines and targeting the immunomodulatory and immunosuppressive actions of endogenous GCs locally within muscles, as reported previously in skin 43. In contrast to the protective action of endogenous GCs in inflammatory muscle wasting *in vivo* in our murine model, we observed effects consistent with muscle wasting *in vitro* in murine primary muscle culture. Consequently, when interpreting *in vitro* studies, it must be recognized that they may not fully reproduce the more complex inflammatory environment conditions seen *in vivo*.

Despite this, given the interest in the therapeutic targeting of 11β‐HSD1 to manage metabolic complications arising from inflammatory diseases, these data provide valuable insights into possible muscle‐related complications.

## Author contributions statement

The authors contributed in the following way: RSH: acquisition of data, data analysis and interpretation, generation of figures, and writing of the manuscript; KR, MSC, GGL: study design, supervision, data analysis, data interpretation, and writing of the manuscript; AF, PMS, CDB: study design and data interpretation; ZH, ML, SAM, MJP, SWJ, AN: acquisition of data and data analysis; all authors: analysis and interpretation of data, and critical revision of the manuscript for important intellectual content.

**SUPPLEMENTARY MATERIAL ONLINE**

**Supplementary figure legends**

**Figure S1.** Levels of mRNA in primary cultures of differentiated myotubes from human quadriceps muscle.

**Figure S2.** Levels of mRNA in in whole tibialis anterior muscle biopsies from mice.

**Table S1.** Joint inflammation scoring system.

**Table S2.** Clinical scoring criteria for determination of systemic inflammatory features in mice.

## Supporting information


**Supplementary figure legends**

**Figure S1** Levels of mRNA in primary cultures of differentiated myotubes from human quadriceps muscle. The levels of mRNA for the differentiation markers (*MYOG, MYOD1, MYF5*), atrophy markers (*FOXO1, FBX32, TRIM63*) and anabolic markers (*IRS1, IGF1, IGF2*) in primary cultures of differentiated myotubes isolated from human quadriceps muscle biopsies determined by RT‐qPCR (n=3 per variable). Human primary cultures were pre‐treated with either vehicle (A‐C, G‐I, M‐O) or, to induce 11β‐HSD1 expression, with TNFβ (10 ng/ml) (D‐F, J‐K, P‐Q) for 48 h prior to a 12 h wash out. Cells were then incubated for 16 h with either vehicle, active cortisol (100 nmol/l) or its inactive precursor cortisone (100 nmol/l) (n=3 per variable). Values are expressed as mean ± standard error. Statistical significance was determined using one‐way ANOVA with a Dunnett's *post hoc* analysis. * p<0.05.

**Figure S2** Levels of mRNA in in whole tibialis anterior muscle biopsies from mice. Levels of mRNA for differentiation markers (*Myog, Myod1*, atrophy markers (*Foxo1, Fbxo32, Trim63*) and anabolic markers (*Igf1, Igf2*) in whole tibialis anterior muscle biopsies isolated from either WT, TNF‐Tg, TNF‐Tg on an 11β‐HSD1KO background and matched 11β‐HSD1KO control mice at 9 weeks (N=6 per group). Values determined by RT‐qPCR and expressed as mean ± standard error. Statistical significance was determined using one‐way ANOVA with a Dunnett's *post hoc* analysis. No significant differences were found.

**Table S1.** Joint inflammation scoring system.
**Table S2.** Clinical scoring criteria for determination of systemic inflammatory features in mice.
