# Supplementary material for: 11β‐Hydroxysteroid dehydrogenase type 1 within muscle protects against the adverse effects of local inflammation
Source: J Pathol. 2016 Oct 18;240(4):472–83. doi: 10.1002/path.4806 (PMC5111591; doi:10.1002/path.4806)
Supplement: Supplementary file 1 — Supplementary figure legends [file PATH-240-472-s004.doc]

**Figure S1.** Levels of mRNA in primary cultures of differentiated myotubes from human quadriceps muscle. The levels of mRNA for the differentiation markers (*MYOG, MYOD1, MYF5*), atrophy markers (*FOXO1, FBX32, TRIM63*) and anabolic markers (*IRS1, IGF1, IGF2*) in primary cultures of differentiated myotubes isolated from human quadriceps muscle biopsies determined by RT-qPCR (n=3 per variable). Human primary cultures were pre-treated with either vehicle (A-C, G-I, M-O) or, to induce 11β-HSD1 expression, with TNFα (10 ng/ml) (D-F, J-K, P-Q) for 48 h prior to a 12 h wash out. Cells were then incubated for 16 h with either vehicle, active cortisol (100 nmol/l) or its inactive precursor cortisone (100 nmol/l) (n=3 per variable). Values are expressed as mean ± standard error. Statistical significance was determined using one-way ANOVA with a Dunnett’s *post hoc* analysis. * *p*<0.05.

**Figure S2.** Levels of mRNA in in whole tibialis anterior muscle biopsies from mice. Levels of mRNA for differentiation markers (*Myog, Myod1*, atrophy markers (*Foxo1, Fbxo32, Trim63*) and anabolic markers (*Igf1, Igf2*) in whole tibialis anterior muscle biopsies isolated from either WT, TNF-Tg, TNF-Tg on an 11β-HSD1KO background and matched 11β-HSD1KO control mice at 9 weeks (N=6 per group). Values determined by RT-qPCR and expressed as mean ± standard error. Statistical significance was determined using one-way ANOVA with a Dunnett’s *post hoc* analysis. No significant differences were found.
