# Supplementary figures and images for: 11β‐Hydroxysteroid dehydrogenase type 1 within muscle protects against the adverse effects of local inflammation
Source: J Pathol. 2016 Oct 18;240(4):472–83. doi: 10.1002/path.4806 (PMC5111591; doi:10.1002/path.4806)

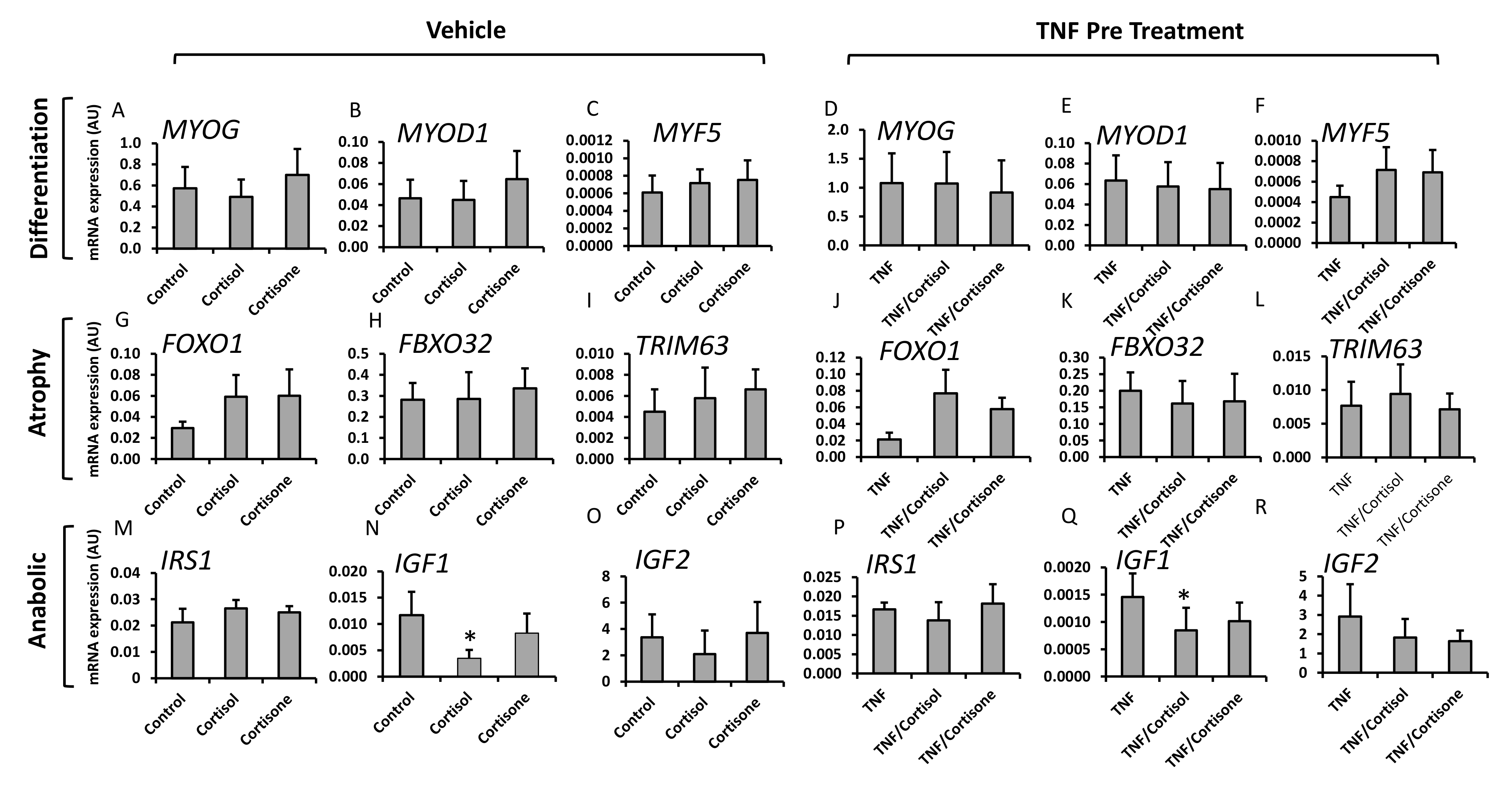

Supplement: Supplementary file 2 — Figure S1 Levels of mRNA in primary cultures of differentiated myotubes from human quadriceps muscle. The levels of mRNA for the differentiation markers (MYOG, MYOD1, MYF5), atrophy markers (FOXO1, FBX32, TRIM63) and anabolic markers (IRS1, IGF1, IGF2) in primary cultures of differentiated myotubes isolated from human quadriceps muscle biopsies determined by RT‐qPCR (n=3 per variable). Human primary cultures were pre‐treated with either vehicle (A‐C, G‐I, M‐O) or, to induce 11β‐HSD1 expression, with TNFβ (10 ng/ml) (D‐F, J‐K, P‐Q) for 48 h prior to a 12 h wash out. Cells were then incubated for 16 h with either vehicle, active cortisol (100 nmol/l) or its inactive precursor cortisone (100 nmol/l) (n=3 per variable). Values are expressed as mean ± standard error. Statistical significance was determined using one‐way ANOVA with a Dunnett's post hoc analysis. * p<0.05. [file PATH-240-472-s001.tif]

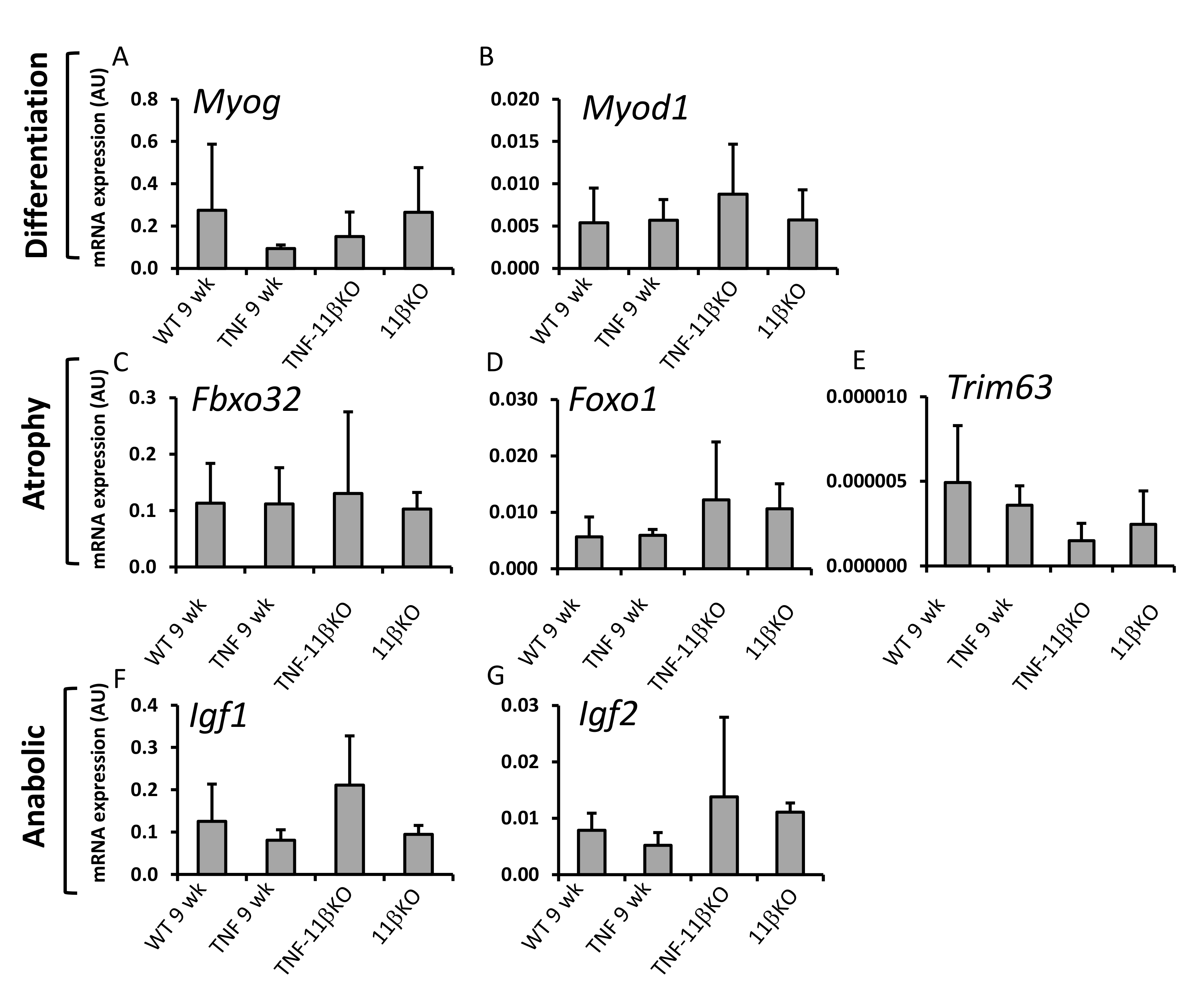

Supplement: Supplementary file 3 — Figure S2 Levels of mRNA in in whole tibialis anterior muscle biopsies from mice. Levels of mRNA for differentiation markers (Myog, Myod1, atrophy markers (Foxo1, Fbxo32, Trim63) and anabolic markers (Igf1, Igf2) in whole tibialis anterior muscle biopsies isolated from either WT, TNF‐Tg, TNF‐Tg on an 11β‐HSD1KO background and matched 11β‐HSD1KO control mice at 9 weeks (N=6 per group). Values determined by RT‐qPCR and expressed as mean ± standard error. Statistical significance was determined using one‐way ANOVA with a Dunnett's post hoc analysis. No significant differences were found. [file PATH-240-472-s003.tif]
