# Supplementary material for: 11β‐Hydroxysteroid dehydrogenase type 1 within muscle protects against the adverse effects of local inflammation
Source: J Pathol. 2016 Oct 18;240(4):472–83. doi: 10.1002/path.4806 (PMC5111591; doi:10.1002/path.4806)
Supplement: Supplementary file 4 — Table S1. Joint inflammation scoring system. Table S2. Clinical scoring criteria for determination of systemic inflammatory features in mice. [file PATH-240-472-s002.doc]

| **Score** | **Signs** |
| --- | --- |
| 1 | Normal |
| 2 | Erythema and mild swelling confined to the hock joint |
| 3 | Erythema and mild swelling extending from the hock or carpus to the metatarsal or metacarpal joints |
| 4 | Erythema and moderate swelling extending from the hock or carpus to metatarsophalangeal or metacarpalophalangeal joints |

**Table S1.** Joint inflammation scoring system. From 5 weeks animals were scored for joint inflammation 3 times a week. Mice were scored using a 16 point system. A single point is awarded for inflammation of any one digit with the maximum score for each limb being 4.

| **Behaviour (assessed in home cage prior to handling)** | |
| --- | --- |
| Normal interactions with cage mates | 0 |
| Reduced interest in roaming behaviour | 2 |
| Isolated from cage mates | 5 |
| **Mobility (assessed in a separate cage)** |  |
| Normal | 0 |
| Abnormal gait | 1 |
| Paddling | 2 |
| Reluctance to stand up on hind legs | 3 |
| Absence of load bearing | 5 |
| **Body weight (compared to an age-matched control)** | |
| Normal (within 10% of age matched control) | 0 |
| >10 % weight loss | 2 |
| >15% weight loss | 5 |
| **Mouse grimace scale** | |
| Not present | 0 |
| Mild | 1 |
| Moderate | 2 |
| **Arthritic paw score (see table right)** | |
| Normal | 0 |
| Total 1 - 3 | 1 |
| Total 4 - 7 | 2 |
| Total 8 - 10 | 3 |
| Total 11 - 12 | 4 |
| **Time since first signs of arthritis were detectable** | |
| 0-1 week | 0 |
| 1-2 weeks | 1 |
| 2-4 weeks | 2 |
| >4 weeks | 4 |
| **Total score** | |
| Sum all parameters | /25 |

**Table S2.** Clinical scoring criteria for determination of systemic inflammatory features in mice. From 5 weeks, mice were scored 3 times weekly. Clinical scores were calculated as the cumulative values from scoring behaviour, mobility, weight loss, mouse grimace, evidence of joint inflammation (Supplementary Table S1) and duration of joint swelling.
